# Description of *Deladenus brevis* n. sp. (Sphaerularioidea: Neotylenchidae) from Iran: a morphological and molecular phylogenetic study

**DOI:** 10.21307/jofnem-2020-062

**Published:** 2020-07-28

**Authors:** Fariba Heydari, Joaquín Abolafia, Majid Pedram

**Affiliations:** 1 Department of Plant Pathology, Faculty of Agriculture, Tarbiat Modares University, Tehran, Iran; 2 Departamento de Biología Animal, Biología Vegetal y Ecología, Universidad de Jaén, Campus Las Lagunillas, s/n, 23071, Jaén, Spain

**Keywords:** *COI* mtDNA, *Deladenus pakistanensis*, Free living mycetophagous phase, LSU rDNA D2-D3, Phylogeny, SSU rDNA, Taxonomy

## Abstract

*Deladenus brevis* n. sp. is described and illustrated based upon its morphological, morphometric, and molecular characters. The new species is mainly characterized by its small body size (367-454 µm long females and 350, 391 µm long males) and has small lateral vulval flaps. It is further characterized by having six lines in the lateral fields, cephalic region slightly expanded, narrower adjacent to the body, short 6 to 7 µm long stylet with three posteriorly sloped knobs, no post-vulval uterine sac (PUS), conical tail, narrowing to a rounded tip, and rare males with slender tylenchoid spicules and bursa reaching the tail tip. By having six lines in the lateral fields, the new species was compared with five known species of the genus, namely *D. apopkaetus, D. cocophilus, D. durus*, *D. obtusicaudatus*, and *D. persicus*, having comparable number (six or seven) of lines in the lateral fields. It was further compared with *D. pakistanensis* having lateral vulval flaps. The differences with above-mentioned species are discussed. In molecular phylogenetic analyses using nearly full length small and large subunit ribosomal DNA (SSU and LSU D2-D3 rDNA) and cytochrome c oxidase subunit I (*COI* mtDNA) gene sequences, *D. brevis* n. sp. formed clade with species of the genus with different clade support values in Bayesian inference.

The genus *Deladenus* was erected by Thorne (1941) with *D. durus* (Cobb, 1922) Thorne (1941) as its type species. Compared to other genera in the family Neotylenchidae (Thorne, 1941), the genus is characterized by its low cephalic region, small stylet, pharyngo-intestinal junction anterior to nerve ring, excretory pore at the level with, or immediately behind the nerve ring, pharynx lacking a median bulb, long overlap of the glands, no post-vulval uterine sac (PUS), and males with bursa. The genus has two entomoparasitic and free-living mycetophagous generations (Siddiqi, 2000). The list of the valid species under the genus is given by several authors (Siddiqi, 2000; Miraeiz et al., 2017; Yu et al., 2017; Morris et al., 2018). Almost all old species under the genus have been established based upon traditional approaches. On the contrary, several recently described species include molecular data (e.g., Yu et al., 2009, 2011, 2013, 2014; Morris et al., 2013; Tomar et al., 2015; Kanzaki et al., 2016). The potential biocontrol ability of *Deladenus siricidicola* (Bedding, 1968) against *Sirex noctilio* Fabricius (Bedding and Akhurst, 1978) has increased the interests on the genus and studying of several aspects of its biology (e.g., Morris et al., 2013). *Deladenus proximus* (Bedding, 1974) appears to be an efficient sterilizer against *Sirex nigricornis* (Zieman et al., 2015). *D. canii* (Bedding, 1974) has been found parasitizing North American *Sirex cyaneus F.* and *Deladenus nevexii* (Bedding, 1974) parasitizes *Xeris spectrum* L. All species of *Deladenus* were assumed to be mycetophagous, until Bedding (1968) found the infective form for some species, showing that two phases occur in their life. Around 13 species of the genus are only known by their mycetophagous phase.

Currently, *D. durus* and *D. persicus* (Miraeiz et al., 2017) are reported from Iran (Jahanshahi Afshar et al., 2014; Miraeiz et al., 2017).

During a survey to recover insect-related nematodes in Iran, a population of an undescribed species of *Deladenus* was recovered from a deadwood sample of a dead forest tree collected from the forests of Golestan province, northern Iran. Thus, the present paper aims to describe the newly recovered species and resolve its phylogenetic relationships with other relevant species and genera using three SSU, LSU rDNA, and *COI* mtDNA markers.

## Materials and methods

### Sampling, nematode extraction, mounting, and drawing

Specimens of *Deladenus brevis* n. sp. were obtained from the bark and rotten wood samples of a dead forest tree collected in Golestan province, northern Iran using the tray method (Whitehead and Hemming, 1965). Live specimens of interested nematodes were handpicked under a Nikon SMZ1000 stereomicroscope, heat killed by adding boiled 4% formalin solution, transferred to anhydrous glycerin, mounted on permanent slides according to De Grisse (1969), and examined using a Nikon Eclipse E600 light microscope. Photomicrographs were taken using an Olympus DP72 digital camera attached to an Olympus BX51 microscope equipped with differential interference contrast. Drawings were made using a drawing tube attached to a microscope and were redrawn using the CorelDRAW® software version 17.

### Scanning electron microscopy (SEM)

Specimens preserved in glycerine were selected for observation under SEM according to Abolafia (2015). They were hydrated in distilled water, dehydrated in a graded ethanol-acetone series, critical point dried, coated with gold, and observed with a Zeiss Merlin microscope (5 kV) (Zeiss, Oberkochen, Germany).

### DNA extraction, PCR, and sequencing

A single live nematode specimen of *D. brevis* n. sp. was picked out and transferred to a small drop of TE buffer (10 mM Tris-Cl, 0.5 mM EDTA; pH 9.0; Qiagen) on a clean slide and squashed using a clean cover slip. The suspension was collected by adding 15 μl TE buffer. The DNA sample was stored at −20°C until used as PCR template. Three DNA samples were prepared in this manner. Primers for LSU rDNA D2-D3 expansion segments amplification were forward D2A (5′-ACAAGTACCGTGAGGGAAAGTTG-3′) and reverse D3B (5′-TCGGAAGGAACCAGCTACTA-3) (Nunn, 1992), forward KKLSU-1 (5′-AAGGATTCCCTTAGTAACGGCGAGTG-3′) (Kiontke et al., 2004), and reverse 1006R (5′-GTTCGATTAGTCTTTCGCCCCT-3′) (Holterman et al., 2008) primers. Primers for partial amplification of SSU rDNA were forward 1813F (5′-CTGCGTGAGAGGTGAAAT-3′) and reverse 1912R (5′-TTTACGGTCAGAACTAGGG-3′) primers (Holterman et al., 2006); forward primer F22 (5′-TCCAAGGAAGGCAGCAGGC-3′) (Dorris et al., 2002) and reverse primer 18 S 1573 R (5′-TACAAAGGGCAGGGACGTAAT-3′) (Mullin et al., 2005). The *COI* mtDNA was amplified using HCO (5′-TAAACTTCAGGGTGACCAAAAAATCA-3′) and LCO (5′-GGTCAACAAATCATAAAGATATTGG-3′) primer pairs (Folmer et al., 1994). The PCR condition for the amplification of genomic fragments and *COI* mtDNA was according to Aliramaji et al. (2020), except that the annealing temperature for *COI* mtDNA amplification was set to 46°C. The PCR products were purified and sequenced directly for both strands using the same primers used in PCR with an ABI 3730 XL sequencer (Bioneer, South Korae). Sequences were deposited in GenBank database under the accession numbers presented in corresponding phylogenetic trees.

### Phylogenetic analyses

The molecular sequences of *D. brevis* n. sp. were compared with those of other nematode species available in GenBank. The previously used sequences for phylogenetic analyses (Tomar et al., 2015; Kanzaki et al., 2016; Yu et al., 2017; Morris et al., 2018) were retrieved from the database, updated by adding extra sequences and the newly generated sequences of the new species. Three independent SSU, LSU, and *COI* mtDNA data sets were prepared. The sequences were aligned using the Q-INS-i algorithm of the online version of MAFFT version 7 (http://mafft.cbrc.jp/alignment/server/) (Katoh and Standley, 2013). The poorly aligned positions and divergent regions of SSU and LSU data sets were eliminated using the online version of Gblocks 0.91b (Castresana, 2000) using all three less stringent options. The *COI* alignment was edited manually using MEGA6 (Tamura et al., 2013). The model of the base substitution was selected using MrModeltest 2 (Nylander, 2004). The Akaike-supported model, a general time-reversible model including among-site rate heterogeneity and estimate of invariant sites (GTR + G + I), was selected for all phylogenetic analyses. Bayesian analysis was performed using MrBayes v3.1.2 (Ronquist and Huelsenbeck, 2003) with a starting random tree and running the chains for four million generations for all. The burn-in phase was set at 25% of the converged runs. The Markov chain Monte Carlo (MCMC) method within a Bayesian framework was used to estimate the posterior probabilities of the phylogenetic tree (Larget and Simon, 1999) using the 50% majority rule. To visualize the results of each run in order to check the effective sample size of each parameter, Tracer v1.5 (Rambaut and Drummond, 2009) was used. The classic rhabditid taxa were selected as outgroups (for species names and accession numbers see trees). The output file of MrBayes was visualized using Dendroscope v3.2.8 (Huson and Scornavacca, 2012) and redrawn in CorelDRAW version 17 (Jahanshahi Afshar et al., 2019a, 2019b).

## Results

### Systematics

**Figure 1:**
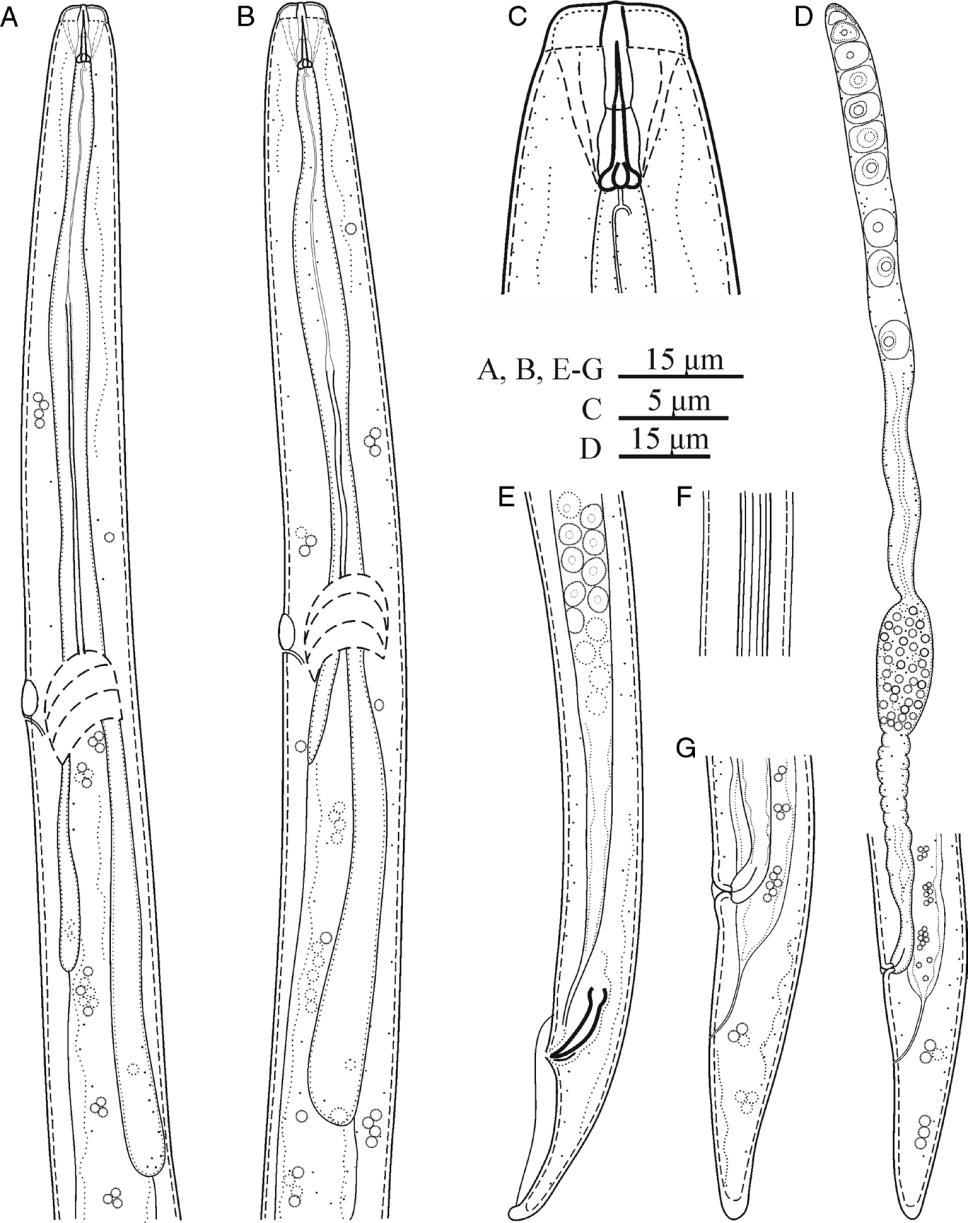
Line drawings of *Deladenus brevis* n. sp. (A, B) Pharynx of male and female; (C) Anterior end of female; (D) Female genital tract and posterior body region; (E) Male posterior body region; (F) Lateral lines; (G) Female tail.

**Figure 2:**
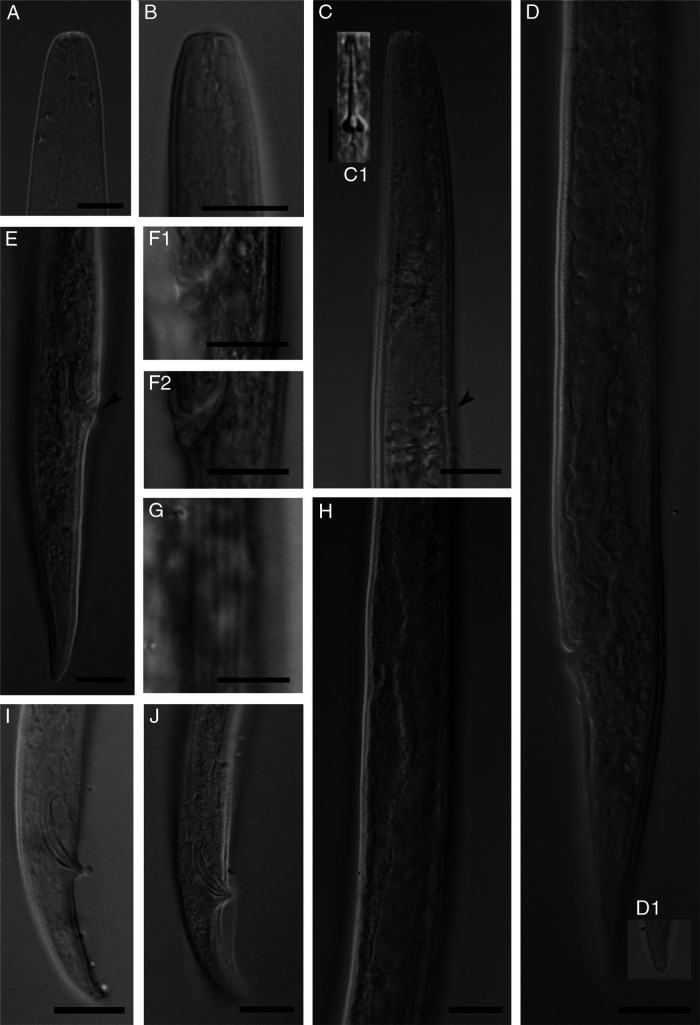
Photomicrographs of *Deladenus brevis* n. sp. (A, B) Anterior body region of female (fresh sample in water and permanently preserved specimen in glycerin respectively); (C) Part of pharynx (arrowhead showing excretory pore and C1 showing details of stylet); (D) Part of female reproductive system; (E) Female posterior body region; (F1 and F2) Lateral vulval flaps in ventrolateral and lateral view in two specimens; (G) Lateral lines; (H) Tip of ovary (arrowhead); (I, J) Male posterior body (Scale bars: A-F2, H-J =10 µm, F1, C1, G =5 µm).

**Figure 3:**
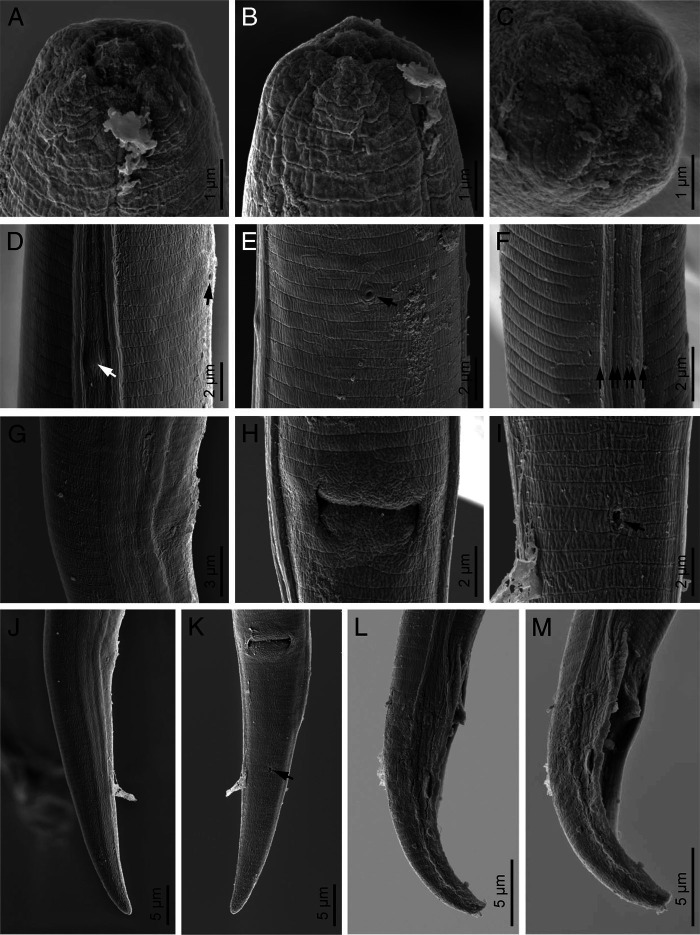
*Deladenus brevis* n. sp. (Scanning Electron Microscopy [SEM]). (A-C) Anterior end in ventral, sublateral and frontal views, respectively. (D) Deirid (white arrow) and excretory pore in lateral view (black arrow). (E) Excretory pore in ventral view (arrow). (F) Lateral field. (G, H) Vulva in lateral and ventral views, respectively. (I) Anus in ventral view (arrow). (J, K) Female posterior end in lateral and ventral views, respectively (arrow pointing the anus). (L, M) Male posterior end in lateral and subventral views, respectively.

*Deladenus brevis* n. sp. (Figs. 1 – 3).

### Measurements

Measurements of the new species are given in Table 1.

**Table 1.** Morphometrics of *Deladenus brevis* n. sp.

	Female		Male
Character	Holotype	Paratypes	Paratypes
*n*	−	8	2
*L*	435	415.5 ± 27.0 (367-454)	391, 350
*a*	26.0	28 ± 3 (22.3-32.3)	35.5, 29.1
*b*	9.5	8.9 ± 0.7 (8.0-10.3)	8.0, 7.6
*c*	15.0	16.8 ± 2.0 (14.3-20.0)	17.7, 15.2
*c′*	3.2	2.8 ± 0.3 (2.4-3.2)	2.7, 2.9
*T* or *V*	88.9	89.0 ± 1.0 (87.2-90.3)	82.3, 85.3
*m*	39.5	39.0 ± 3.0 (33.3-43.1)	50.9, 46.3
Cephalic region height	1.4	1.9 ± 0.2 (1.5-2.1)	2.0, 1.5
Cephalic region width	5.7	5.9 ± 0.4 (5.0-6.5)	5.2, 6
Max. body dim.	17	15 ± 2 (13-18)	11, 13
Stylet total length	6.6	6.4 ± 0.3 (6-7)	6.0, 6.5
Stylet conus	2.6	2.5 ± 0.2 (2-3)	2.2, 2.5
Excretory pore from anterior	78	79.0 ± 5.3 (67-86)	75.0, 70.5
Nerve ring from anterior	71	71.5 ± 9.0 (60-83)	52, ?
Hemizonid from anterior	76	77.5 ± 6.0 (66-86)	72, 69
Vulval body diameter	13	12.5 ± 2. (10-18)	−
Anal body diameter	9	9.0 ± 1.2 (7-10)	8, 9
Tail	29	25.0 ± 3.4 (20-30)	22, 23
Spicules	−	−	11.3, 14.5
Gubernaculum	−	−	3.4, 4.5

**Note:** All measurements are in μm and in the form: Mean ± SD (range).

### Free-living mycetophagous female

Females are small-sized nematodes, with slender body, gradually narrowing toward posterior end by having a conical tail. Lateral fields are with six lines. Cephalic region is low, slightly narrower than the adjacent body in permanent mounts. Stylet is small, with three small posteriorly sloping knobs (the knobs and general shape of stylet well visible in fresh material in temporary slides). Dorsal gland orifice is just posterior to stylet knobs. Hemizonid is at the level with nerve ring. Excretory pore is at the level with, or immediately behind, hemizonid. Deirid is slightly anterior to excretory pore. Pharynx is with no median bulb, lacking a chamber, slightly wider at pharyngo-intestinal junction, the latter anterior to nerve ring, and 42 to 52 µm from anterior end. Dorsal gland is long, with overlapping intestine. Intestine is simple, and the rectum and anus are functional. The reproductive system is monodelphic-prodelphic, anteriorly outstretched, composed of an ovary with maturing oocytes at single row, tubular oviduct, axial large ellipsoid spermatheca with fine spheroid sperm, crustaformeric with further than four cells in each of the four rows, uterus, vagina with moderately sclerotized wall, no PUS, and vulva with small lateral flaps. One or two eggs were observed in uterus of mature females. Tail is conical, narrowing to a rounded tip.

### Infective female

Not found.

### Parasitic female

Not found.

### Free-living mycetophagous male

Rare: general morphology and pharynx are similar to those of female. Genital system is monorchic. They have slender spicules tylenchoid. Gubernaculum is thin and small. Bursa reaches tail terminus.

### Type habitat and locality

Specimens were recovered from the wood and bark samples of a dead broadleaf forest tree, collected in Golestan province, northern Iran, during October 2019. GPS coordinates were 36°44′57.5″ N, 54°19′ 12.0″ E.

### Type specimens

Holotype female, seven paratype females, and one paratype male were deposited at USDA nematode collection (five slides with accession codes T-7475p to T-7479p) (one paratype female and one paratype male were used for SEM preparations). The LSID code of this publication is: urn: lsid:zoobank.org:pub: 34D40A8F-A13A-473C-BB24-E8AEB702EDA9.

### Etymology

The specific epithet refers to the short body of the new species.

### Differential diagnosis

*Deladenus brevis* n. sp. (the mycetophagous phase) is delimited in the genus by its small body size and small lateral vulval flaps. It is further characterized by six lines in the lateral fields, short 6 to 7 μm long stylet with three posteriorly sloping basal knobs well visible in fresh material in water and rare males. By having six lines in the lateral fields, the new species is comparable with five known species of the genus, namely *D. apopkaetus* (Chitambar, 1991), *D. cocophilus* (Nasira et al., 2013), *D. durus*, *D. obtusicaudatus* (Bajaj, 2015), and *D. persicus* having a comparable number of lines in the lateral fields. By having lateral vulval flaps, the new species was also compared with *D. pakistanensis* (Shahina and Maqbool, 1992). The comparisons of the new species with six aforementioned species are given as follows.

From *D. apopkaetus* by shorter females (415.5 (367-454) vs 743 (612-840) μm), excretory pore at the level with, or immediately behind hemizonid (vs anterior), lacking a median pharyngeal chamber (vs present), shorter stylet (6.4 (6-7) vs 10.9 (8.7-13.5) μm), anteriorly located vulva (*V* = 89 (87.2-90.3) vs 91 (90-93)) and shorter tail (25 (20-30) vs 37 (31-43) μm).

From *D. cocophilus* by shorter females (415.5 (367-454) vs 740 (550-930) µm), shorter stylet (6.4 (6-7) vs 8.9 (8-10) μm), vulval lips not protuberant and body not abruptly narrowing at posterior of vulva (vs vulval lips protuberant with sharp posterior constriction of the body after vulva).

From *D. durus* (the data of *D. durus* after Chitambar, 1991) by lacking a median pharyngeal chamber (vs present) and shorter females (415.5 (367-454) vs 796-1360 µm).

From *D. obtusicaudatus* by shorter females (415.5 (367-454) vs 730 μm), excretory pore at the level with, or immediately behind hemizonid (vs anterior), smaller *c* (16.8 (14.3-20.0) vs 29.0-29.3), greater *c′* (2.8 (2.4-3.2) vs 1.5-1.7), anteriorly located vulva (*V* = 89 (87.2-90.3) vs 92.6-93.5), shorter stylet (6.4 (6-7) vs 9 µm) and female tail shape (conical, narrowing to a rounded tip vs cylindrical with broadly rounded to truncated terminus).

From *D. persicus* by shorter body (415.5 (367-454) vs 548 (474-609) μm) not remarkably narrowing posterior to vulva (vs narrowing), lacking a median pharyngeal chamber (vs present) and anteriorly located vulva (*V* = 89 (87.2-90.3) vs 90.3 (92.0-93.7)).

From *D. pakistanensis* by its body not abruptly narrowing at posterior of vulva (vs body abruptly narrowing posterior to vulva), shorter females (415.5 (367-454) vs 719 (636-761) μm), number of lines in lateral fields (6 vs 10-12) and anteriorly located vulva (*V* = 89 (87.2-90.3) vs 92 (92-93)).

### Molecular profiles and phylogenetic status

#### Nearly full length SSU rDNA phylogeny

**Figure 4:**
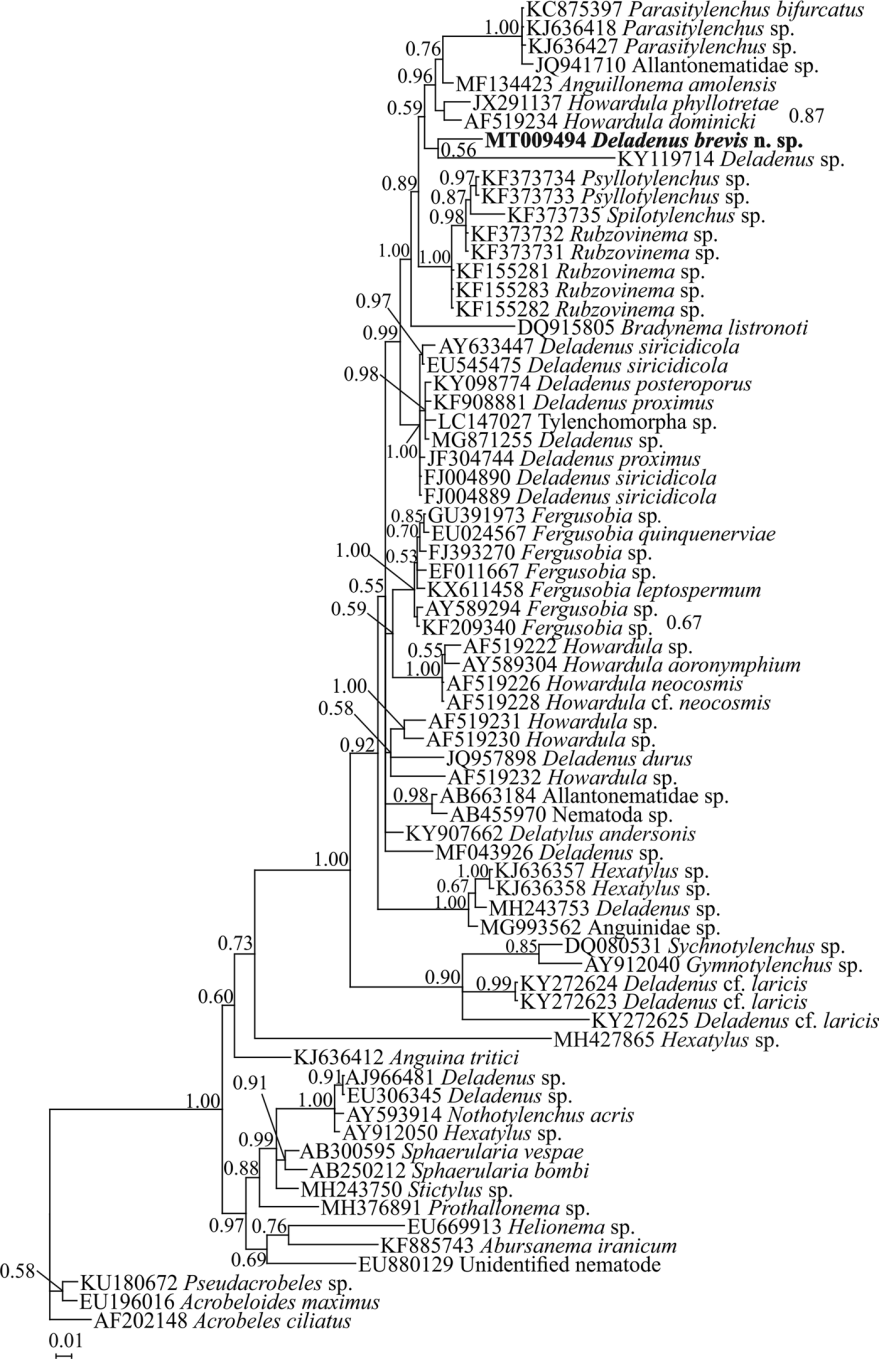
Bayesian 50% majority rule consensus tree of *Deladenus brevis* n. sp. based on SSU rDNA sequences under GTR + I + G model. Bayesian posterior probability values more than 0.50 are given for appropriate clades. The new sequence is indicated in bold.

To determine the phylogenetic relationships of *D. brevis* n. sp. with other species, a newly obtained 1674 nt nearly full length SSU rDNA sequence with accession number MT009494 was used. The BLAST search using this fragment revealed that it is unique, and its identity with other previously submitted sequences to the database is less than 96%. A number of 71 sphaerularid sequences (including one sequence of the new species) and sequences of three classic rhabditids as outgroup taxa (species names and accession numbers in SSU tree) were used in SSU phylogeny. The SSU data set was composed of a total of 1,611 characters, of which 562 characters were variable, with an average nucleotide composition of 25.6% A, 20.9% C, 26.8% G, and 26.7% T. Figure 4 represents the Bayesian phylogenetic tree inferred using this data set. In this tree, the new species is in poorly supported sister relation with *Deladenus* sp. (KY119714). The polyphyletic nature of *Deladenus* is seen in this tree.

### D2 to D3 fragments of LSU rDNA phylogeny

**Figure 5:**
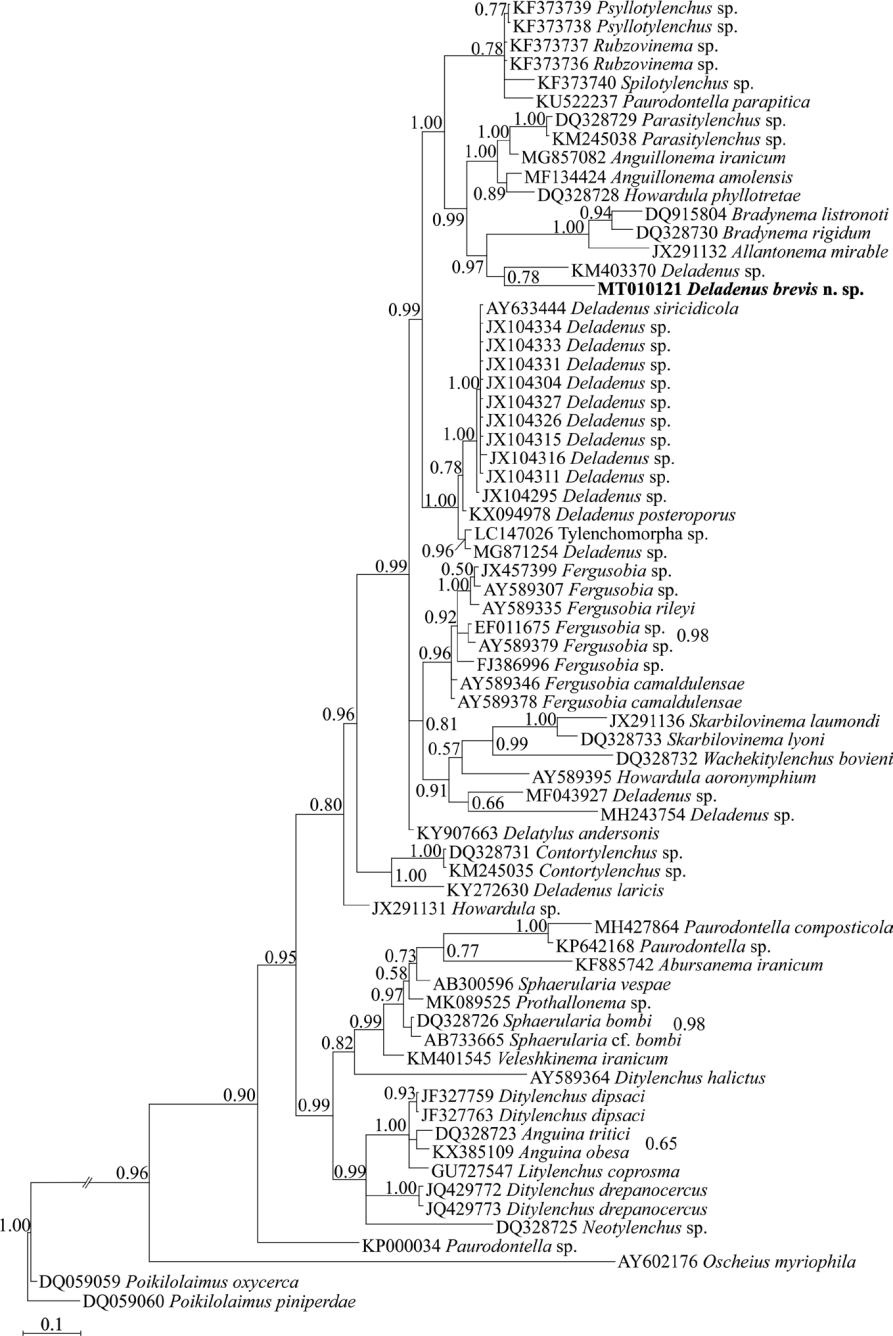
Bayesian 50% majority rule consensus tree of *Deladenus brevis* n. sp. based on LSU rDNA D2 to D3 sequences under GTR + I + G model. Bayesian posterior probability values more than 0.50 are given for appropriate clades. The new sequence is indicated in bold.

To reconstruct the LSU rDNA tree, the newly obtained 882 nt long sequence of D2 to D3 expansion segments of LSU rDNA with accession number MT010121 was used. The BLAST search using this sequence revealed that its identity with other previously submitted sequences to the database is less than 86%. A number of 70 sphaerularid sequence and sequences of two classic rhabditids as outgroup taxa (including one sequence of the new species) were used for inferring the LSU phylogeny. The LSU alignment was composed of 447 total characters, of which 281 characters were variable, with an average nucleotide composition of 25.8% A, 20.3% C, 31.5% G, and 22.4% T. Figure 5 represents the phylogenetic tree inferred using this data set. In this tree, the new species is in sister relation with an unidentified species of the genus (KM403370) with moderate support in Bayesian inference. *Deladenus* appeared non-monophyletic in this tree.

### *COI* mtDNA phylogeny

**Figure 6.**
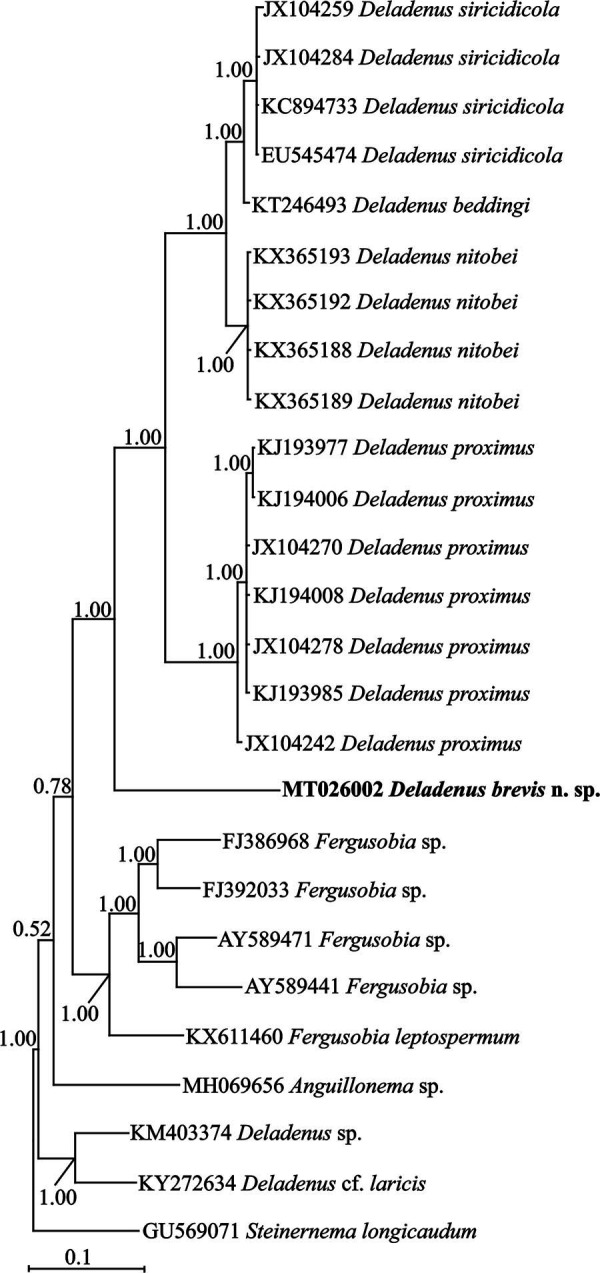
Bayesian 50% majority rule consensus tree of *Deladenus brevis* n. sp. based on *COI* mtDNA sequences under GTR + I + G model. Bayesian posterior probability values more than 0.50 are given for appropriate clades. The new sequence is indicated in bold.

To reconstruct the *COI* tree, a newly obtained 634 nt long sequence of *COI* mtDNA with the accession number MT026002 was used. The BLAST search using this sequence revealed that its identity with other previously submitted sequences to the database is less than 81%. A number of 28 sphaerularid sequences (including one sequence of the new species) and one sequence of a classic rhabditid as outgroup taxon were used for inferring the *COI* phylogeny. The *COI* alignment was composed of 651 total characters, of which 262 characters were variable, with an average nucleotide composition of 21.0% A, 11.8% C, 20.7% G, and 46.5% T. Figure 6 represents the phylogenetic tree inferred using this data set. In this tree, the new species is in sister relation with a major clade including four species of *Deladenus.* Two other isolates of the genus (MK403374 and KY272634) are distantly related to the aforementioned clade (the new species + four other species), and based on this tree, the genus is not monophyletic.

## Discussion

In present study, a population of the genus *Deladenus*, representing a new member to it, was recovered and described from Iran. Previously, two species of the genus had been reported from the country (Jahanshahi Afshar et al., 2014; Miraeiz et al., 2017). The newly described species in this study has a small body size, small lateral vulval flaps and by these features, it is well delimited in the genus. Previously, vulval flap was only reported for *D. pakistanensis* (Shahina and Maqbool, 1992). Most species of the family Neotylenchidae are insect associates (Siddiqi, 2000) with the exception of a few *Deladenus*.

Currently, molecular data are only available for a limited number of identified *Deladenus* spp., namely *D. siricidicola* (Yu et al., 2009; Mlonyeni et al., 2011; Leal et al., 2012; Morris et al., 2013), *D. proximus* (Yu et al., 2011; Morris et al., 2013; Zieman et al., 2015; Hartshorn et al., 2017), *D. prorsus* (Yu et al., 2013), and *D. nitobei* (Kanzaki et al., 2016). Several old species under the genus are poorly described and their type materials are hardly accessible. An integrative taxonomic study including both traditional and molecular data and species of close genera is recommended in taxonomic studies of this group of nematodes. In the present study, currently available sequences of two close genera *Delatylus* (Yu et al., 2018) and *Hexatylus* (Goodey, 1926) were also included. Currently, only few sequences were available for the genus *Hexatylus*, a close genus to *Deladenus*, and the morphological data of the currently sequenced populations were also inaccessible. Besides nonmonophyletic nature of most sphaerularid genera (Mobasseri et al., 2017, present study), the molecular data were useful in identification purposes at species level.

In presently resolved phylogenies using two genomic and one mitochondrial markers, the non-monophyly (polyphyletic, using SSU and LSU, and paraphyletic, using *COI* mtDNA) of *Deladenus* was observed.
